# Implementation of an epicardial implantable MEMS sensor for continuous and real-time postoperative assessment of left ventricular activity in adult minipigs over a short- and long-term period

**DOI:** 10.1063/5.0169207

**Published:** 2024-04-16

**Authors:** C. Zinno, F. Agnesi, G. D'Alesio, A. Dushpanova, L. Brogi, D. Camboni, F. Bernini, D. Terlizzi, V. Casieri, K. Gabisonia, L. Alibrandi, C. Grigoratos, J. Magomajew, G. D. Aquaro, S. Schmitt, P. Detemple, C. M. Oddo, V. Lionetti, S. Micera

**Affiliations:** 1 The BioRobotics Institute, Department of Excellence in Robotics & AI, Scuola Superiore Sant'Anna, Pisa, Italy; 2 Unit of Translational Critical Care Medicine, Laboratory of Basic and Applied Medical Sciences, Interdisciplinary Research Center “Health Science,” Scuola Superiore Sant'Anna, Pisa, Italy; 3 Al-Farabi Kazakh National University, Almaty, Kazakhstan; 4 Bio@SNS, Scuola Normale Superiore, Pisa, Italy; 5 BioMedLab, Interdisciplinary Research Center “Health Science,” Scuola Superiore Sant'Anna, Pisa, Italy; 6 Fondazione Toscana “G. Monasterio,” Pisa, Italy; 7 Department of Chemistry, Fraunhofer Institute for Microengineering and Microsystems, 55129 Mainz, Germany; 8 University of Pisa, Pisa, Italy; 9 Institute of Clinical Physiology, National Council of Research, Pisa, Italy; 10 Bertarelli Foundation Chair in Translational NeuroEngineering, Centre for Neuroprosthetics and Institute of Bioengineering, École Polytechnique Fédérale de Lausanne (EPFL), Lausanne, Switzerland

## Abstract

The sensing of left ventricular (LV) activity is fundamental in the diagnosis and monitoring of cardiovascular health in high-risk patients after cardiac surgery to achieve better short- and long-term outcome. Conventional approaches rely on noninvasive measurements even if, in the latest years, invasive microelectromechanical systems (MEMS) sensors have emerged as a valuable approach for precise and continuous monitoring of cardiac activity. The main challenges in designing cardiac MEMS sensors are represented by miniaturization, biocompatibility, and long-term stability. Here, we present a MEMS piezoresistive cardiac sensor capable of continuous monitoring of LV activity over time following epicardial implantation with a pericardial patch graft in adult minipigs. In acute and chronic scenarios, the sensor was able to compute heart rate with a root mean square error lower than 2 BPM. Early after up to 1 month of implantation, the device was able to record the heart activity during the most important phases of the cardiac cycle (systole and diastole peaks). The sensor signal waveform, in addition, closely reflected the typical waveforms of pressure signal obtained via intraventricular catheters, offering a safer alternative to heart catheterization. Furthermore, histological analysis of the LV implantation site following sensor retrieval revealed no evidence of myocardial fibrosis. Our results suggest that the epicardial LV implantation of an MEMS sensor is a suitable and reliable approach for direct continuous monitoring of cardiac activity. This work envisions the use of this sensor as a cardiac sensing device in closed-loop applications for patients undergoing heart surgery.

## INTRODUCTION

The proportion of patients undergoing different types of elective cardiac surgery and heart transplantation has increased over time, especially in the older population.^1^ Despite accounting for less than 15% of in-patient procedures, high-risk patients account for 80% of deaths.^2^ Improved postoperative outcome remains an important determinant of functional recovery, long-term survival, and healthcare costs.^3^ The excitation-contraction coupling in the heart is a fundamental process that ensures proper cardiac function across different heart rates and contributes to the regulation of hemodynamics. This process is primarily influenced by the autonomic nervous system.^4^ The cardiac contractility is closely related to the HR in the presence of normal electro-mechanical coupling.^5^ As a result, it is also possible a reliable direct estimation of the heart rate from beating motions in the absence of arrhythmias.^6^ In addition to heart rate, left ventricle activity monitoring may be crucial to detect systolic and diastolic dysfunctions, up to the onset of heart failure (HF).^7–9^ So far, the left ventricular pressure (LVP) is the gold standard measure used for the diagnosis of diastolic dysfunction. It is a key player in the pathophysiology of HF, having prognostic implications even in the preclinical stage.^9^ The more accurate procedure to obtain information about left ventricle activity is the heart cathetherization,^10,11^ but the consequences of its invasiveness and the risk to use it as a routine tool cannot be underestimated.^12^ Conventional noninvasive methods such as electrocardiography, photoplethysmography, and sphygmomanometry have shown limited efficacy in reducing mortality and hospitalization,^13–15^ having significant confounding factors.^16^ By supplementing ECG analysis and blood pressure monitoring, the direct assessment of cardiac movements during contraction and relaxation cycles has demonstrated its potential in preventing sudden cardiac death.^17^ Implantable cardiac sensors, being in closer contact with the heart, offer advantages in terms of increase in precision, higher signal-to-noise ratio (SNR), and the ability to detect slight variations in cardiac activity.^18^ Currently, the challenges are represented by biocompatibility, minimization of invasiveness, and measurement reliability.^18–20^ Different technologies are employed to invasively measure cardiac activity through monitoring of related physiological variables, as implantable cardiac monitors, pressure sensors, accelerometers, impedance sensors, and strain sensors.^21–27^

MEMS technology has emerged as a valuable solution in the field of invasive cardiac sensing, thanks to its unique properties. Indeed, MEMS have become ubiquitous in the last 20 years, remodeling the industry of healthcare.^28–31^ MEMS sensors can be easily fabricated with mm/*μ*m-scale with standard microfabrication techniques from the integrated circuits industry, typically starting from silicon or glass substrates. Their compact size offers the possibility of performing minimally invasive surgical procedure, minimizing damage to surrounding organs, and enabling precise anatomical placement.^30^ To ensure long-term biocompatibility and reliability, these devices are typically encapsulated in biocompatible polymers^32^ [e.g., polydimethylsiloxane (PDMS), polyimide, and parylene], reducing the mechanical and chemical mismatch with the tissues. Additionally, MEMS technology facilitates multi-modal sensing within a single device, enabling sustained simultaneous measurement of various physiological variables, such as pressure and strain.^33^ This comprehensive acquisition of different mechanical information provides a complete view of cardiac activity, improving follow-up and timing of treatment of high-risk patients. In research and clinical scenarios, implantable MEMS sensors have been employed for short-term applications in animals and patients.^34–38^ As an example, the short-term reliability of a cardiac sensor based on a commercial MEMS 3-axis accelerometer was evaluated in pigs.^39^ To ensure biocompatibility of the device, a flexible polyimide connecting cable was covered by silicone, and the sensor was encapsulated in a 3D-printed metal structure. During external pacing sessions, the device underwent testing to record the three-axis acceleration of the myocardial surface. Moreover, a wireless MEMS pressure sensor has been implanted on the aortic wall of mice, obtaining a down-scaled recording of the blood pressure waveform.^40^ The device was composed of a silicone cuff filled by biocompatible, insulating fluid where a MEMS pressure sensor is immersed, together with integrated electronics. The integration of the MEMS sensor with the readout and powering integrated circuit had a size of 2.2 × 2.2 mm^2^. However, failures in the packaging led to 3 dB noise increase after the implant, which negatively impacted the performance of the sensor. Although short-term studies have exhibited promising outcomes, there is a requirement for long-term evaluation of implantable cardiac MEMS sensors as a means of cardiovascular monitoring. Some clinical trials have shown effective long-term performances of cardiac MEMS sensors in monitoring pulmonary arterial pressure and in the early detection of the onset of heart failure.^41–44^ All the studies have been performed using the CardioMEMS^®^ device, a MEMS capacitive sensor for pulmonary artery pressure measurement. The working principle is based on a change of the resonant frequency of its electrical circuit in response to applied pressure.^45^ The CHAMPION (CardioMEMS Heart Sensor Allows Monitoring of Pressure to Improve Outcomes in NYHA Class III Heart Failure Patients) clinical trial demonstrated significant positive results, with a notable 33% reduction in hospitalization rates and a 23% reduction in mortality. The effectiveness of MEMS cardiac sensors for cardiovascular disease (CVD) monitoring in improving patient outcome was reinforced by the implementation of a subsequent clinical trial, known as MEMS-HF (CardioMEMS European Monitoring Study for Heart Failure). These findings highlight the potential of MEMS cardiac sensors as valuable tools for life-saving closed-loop applications, enabling continuous monitoring of cardiovascular function in high-risk patients. Examples of such devices include cardiac resynchronization therapies and implantable cardioverter-defibrillators.^46^

In our study, we present an implantable MEMS sensor fabricated using standard photolithographic techniques and encapsulated in medical-grade silicone, designed to sense left ventricular (LV) activity. The sensor design, shape, and dimensions have been optimized to match the LV wall. During the implantation procedure, the sensor is sutured onto the epicardium of the LV with a pericardial patch graft. To validate and characterize the sensor, we conducted benchtop tests using a silicone phantom of the left ventricle. Subsequently, we performed short- and long-term studies using a healthy adult male minipig, which is a suitable species for cardiovascular research.^47^ The results demonstrated consistent and reliable monitoring of cardiac activity even after 30 days of implantation. Histological analyses of the LV implantation site after sensor retrieval shown no sign of myocardial fibrotic response. In addition, we were able to obtain intraventricular information from the epicardial sensor, comparing our results with previous studies using LV cathetherization for the evaluation of left ventricular end pressure (LVEDP).^48,49^ These findings underscore the potential of this technology as a sensor for closed-loop applications in the follow-up high-risk cardiac surgical patients.

## RESULTS

### Benchtop characterization

**FIG. 1.**
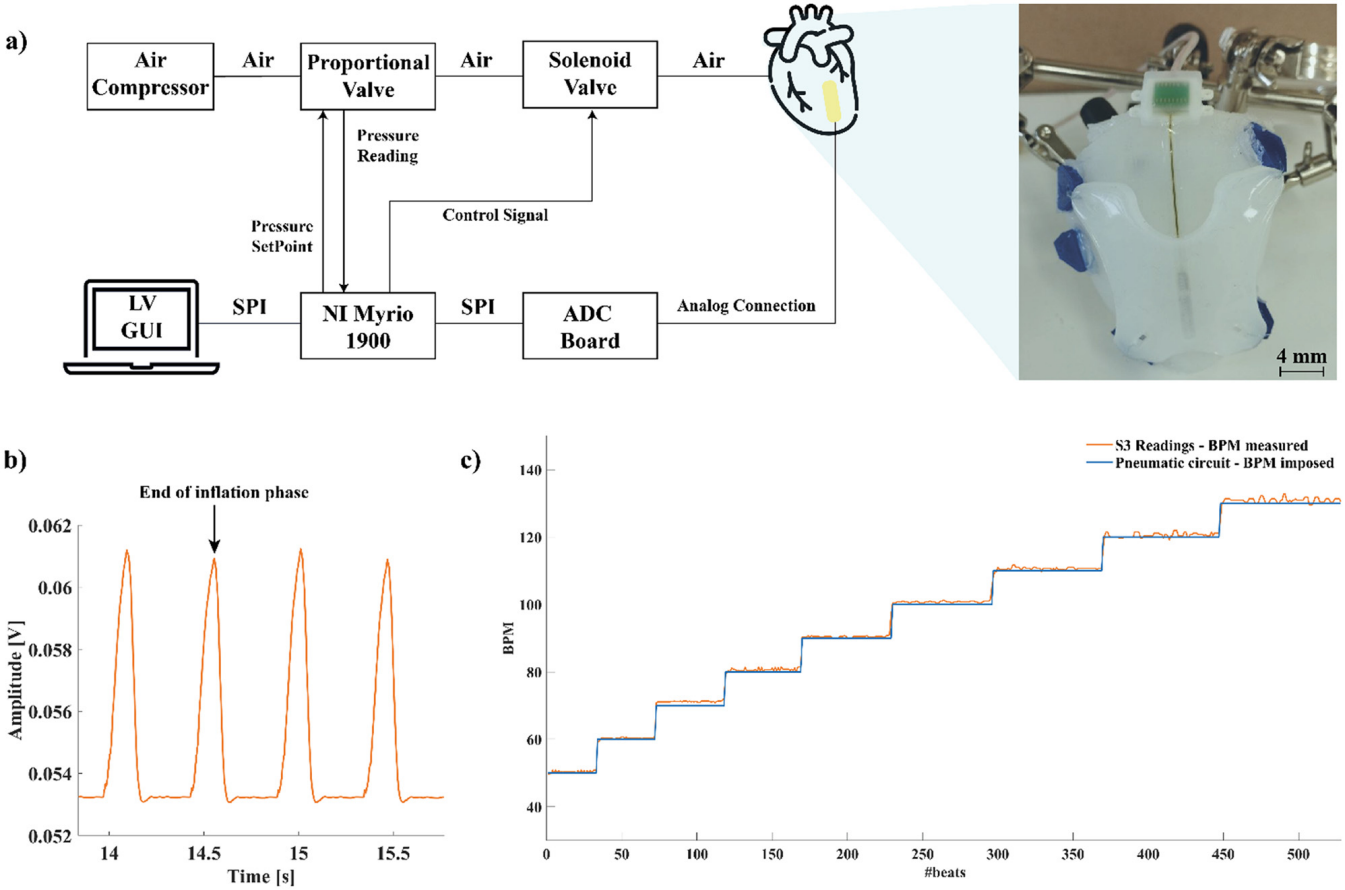
(a) Pneumatic circuit schematic (left) with silicone phantom magnification (right). The air inflation/deflation cycle is controlled by a solenoid valve, allowing the air flow from the air compressor to the ventricle silicone phantom. The control signal is delivered by a NI MyRIO 1900 driven by a custom designed graphical user interface (GUI) in LabVIEW (LV). The MEMS sensor is kept fixed on the phantom, thanks to a polymer hodge sutured to four eyelets (right sub-figure, in blue) to guarantee mechanical stability. Data are acquired through the ADC board. (b) MEMS S3 channel output. The signal has a single peak representing the end of the inflation phase, followed by a sharp decrease indicating air deflation. (c) BPM sweep at a fixed inflation pressure (120 mbar) shows the capability of the sensor to follow HR variations with minimal errors.

We analyzed benchtop data to retrieve information about the ability of the sensor to record heartbeats. First, we evaluated the shape of the MEMS signal [Fig. 1(b)] in response to the inflation/deflation cycle of the silicone phantom. The MEMS signal has a single peak representing the moment of maximal deformation of the phantom, at the end of the inflation phase, after which there is a steep decrease due to the deflation phase. We compared the HR obtained from the MEMS sensor to the HR imposed by the pneumatic circuit by computing the RMSE, used to evaluate the reliability of the MEMS sensor in computing the HR comparing it to a reference signal. The results of the benchtop characterization, summarized in Table I, together with Fig. 1(c) highlight the capability of the sensor to reliably obtain HR information. Th/e results of the single characterization tests for all MEMS sensor channels are shown in the Supplementary Material (Tables S1–S9).

**TABLE I.** Benchtop characterization results (n = 9 steps performed in the BPM sweep) are presented as mean ± std.

#MEMS sensor	Imposed BPM	Imposed pressure (mbar)	Duty cycle (%)	RMSE
S1	50:10:130	80	30	1.7582 ± 1.1084
S1	50:10:130	100	30	1.2833 ± 0.5908
S1	50:10:130	120	30	1.4989 ± 0.8856
S2	50:10:130	80	30	1.1675 ± 0.3389
S2	50:10:130	100	30	1.0375 ± 0.4673
S2	50:10:130	120	30	1.1737 ± 0.5440
S3	50:10:130	80	30	1.1060 ± 0.5360
S3	50:10:130	100	30	1.0528 ± 0.8742
S3	50:10:130	120	30	0.9849 ± 0.3801

### Results of acute *in vivo* experiments

**FIG. 2.**
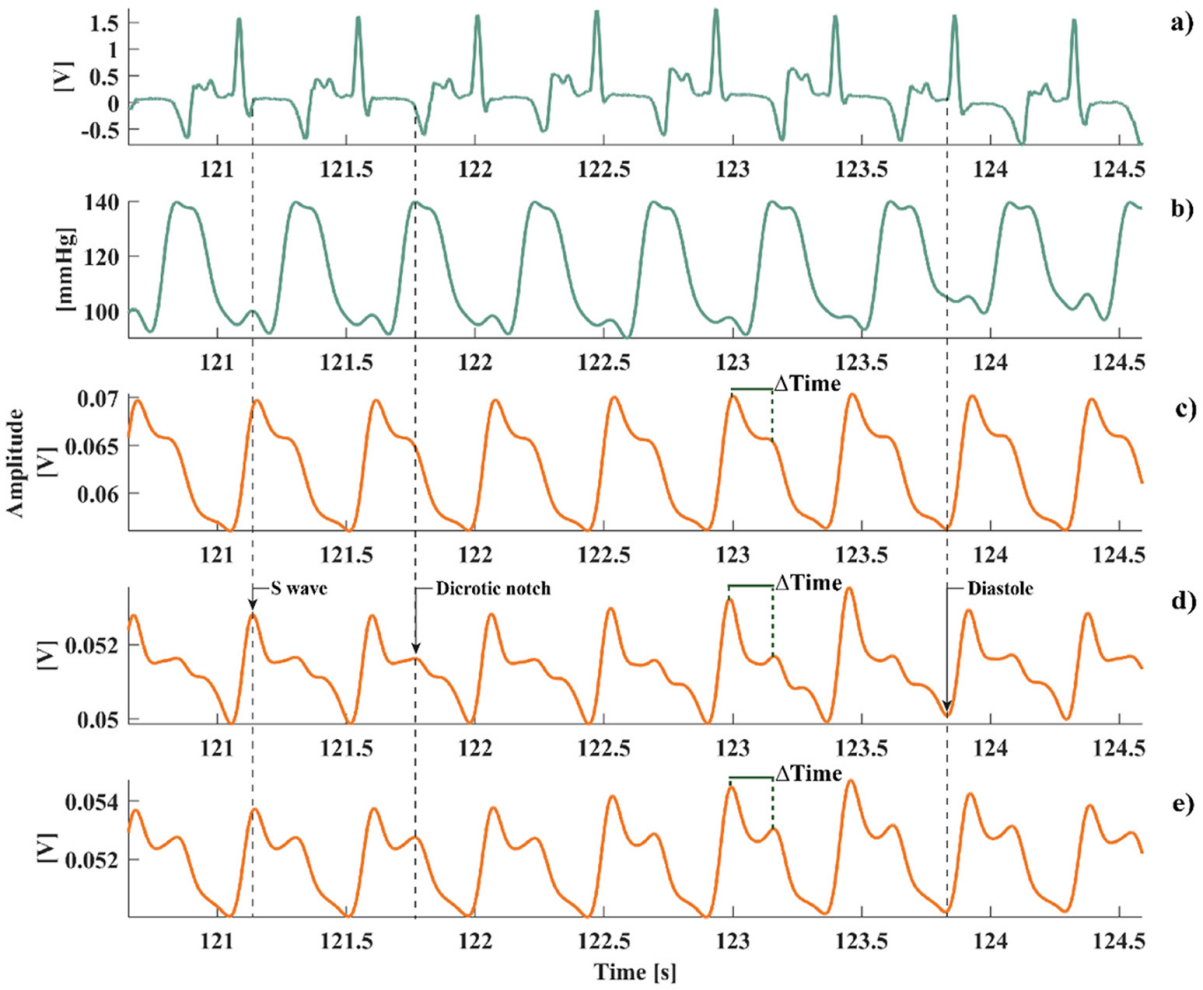
Comparison between ECG, ABP signal, and MEMS S1–S3 channels. (a) Low-pass filtered (moving average filter, n = 15 samples) ECG signal. (b) Low-pass filtered (10 Hz cutoff frequency) arterial BP signal. (c) S3 channel. (d) S2 channel. (e) S1 channel. The shape of S1/S3 channels is similar to the ABP signal and coherent with the ECG: the first positive peak corresponds to the S wave, representing ventricular depolarization, then there is a smoother peak (indicating the dicrotic notch), and then the decrease up to a plateau indicating the diastole. The dashed line indicates the corresponding phases in the ECG and ABP signals. In the S2 channel, also a third smoother peak can be observed. In each of the channel, we evaluated ΔTime as the distance between the first two peaks of the waveform.

In Fig. 2, the filtered ABP and ECG signal shape [Fig. 2(a)] were compared to the filtered MEMS signal shapes from the three channels. S1 [Fig. 2(b)] and S3 [Fig. 2(d)] shapes present a pattern with a first sharp peak aligned with the S wave, corresponding to the left ventricle depolarization followed by a valley and a smoother peak (dicrotic notch) and then a decrease in the signal up to a minimum (diastolic peak). The S2 [Fig. 2(c)] shape is different compared to S1 and S3 in the presence of a third smoother peak. Nevertheless, it is always possible to detect the systolic and diastolic peaks, necessary for HR computation, also from S2.

To evaluate the MEMS sensor's *in vivo* performances, we analyzed 1-min signal recordings (n = 6) for each acute session. Then, we computed the HR using the signal obtained from the sensor, and we compared it to the HR computed through the ABP signal (used as a reference). The comparison was carried out by computing the RMSE. For two acute sessions (AS1 and AS3), we obtained an RMSE lower than 1 BPM considering all three MEMS sensor channels (Table II), while for AS2, both the mean and the standard deviation (std) are close to 1 BPM for all three channels. The discrepancy in the results can be attributed to a deterioration of the signal quality during AS2 related to issues in the connector linking the sensor and the ADC system.

**TABLE II.** MEMS sensor *in vivo* characterization in acute sessions. RMSE was computed between the MEMS signal and the ABP in 1-min recordings (n = 6) during acute sessions. Results are presented as mean ± std.

#Session	Recording duration (min)	#Sensor	RMSE
AS1	1	S1	0.0692 ± 0.0104
AS1	1	S2	0.0712 ± 0.0105
AS1	1	S3	0.0711 ± 0.0133
AS2	1	S1	1.6017 ± 1.3669
AS2	1	S2	0.9836 ± 0.6821
AS2	1	S3	1.6940 ± 1.1118
AS3	1	S1	0.5669 ± 0.4143
AS3	1	S2	0.1346 ± 0.1105
AS3	1	S3	0.0580 ± 0.0112

**FIG. 3.**
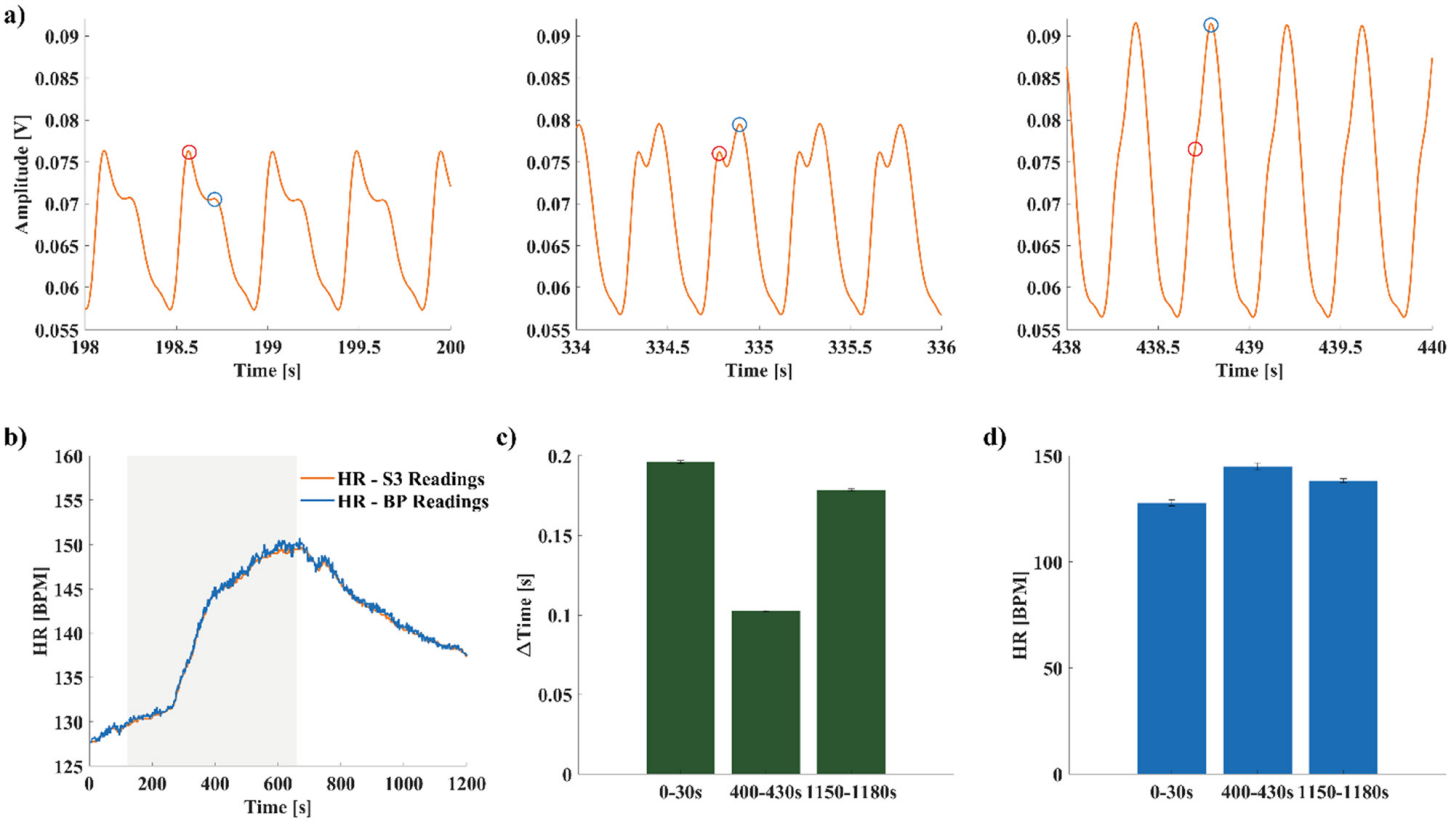
Dobutamine pharmacological challenge. (a) S3 channel readings during different intervals of the pharmacological challenge. At baseline conditions, the second peak (blue circle) is lower than the first one (red circle) representing the systole, then with increasing HR, it becomes larger overcoming the first one, increasing its amplitude. (b) HR computed using the S3 channel and the BP readings. The gray shaded area represent the duration of the dobutamine injection. (c) ΔTime variations during 30 s windows in three different time intervals: 0–30 s (baseline), 400–430 s (HR minimum), and 1150–1180 s (return to baseline). (d) HR variations during 30 s windows in three different time intervals: 0–30 s (baseline), 400–430 s (HR minimum), and 1150–1180 s (return to baseline). As for the β-blockers challenge, we observed an inversely proportional relationship between ΔTime and HR.

**FIG. 4.**
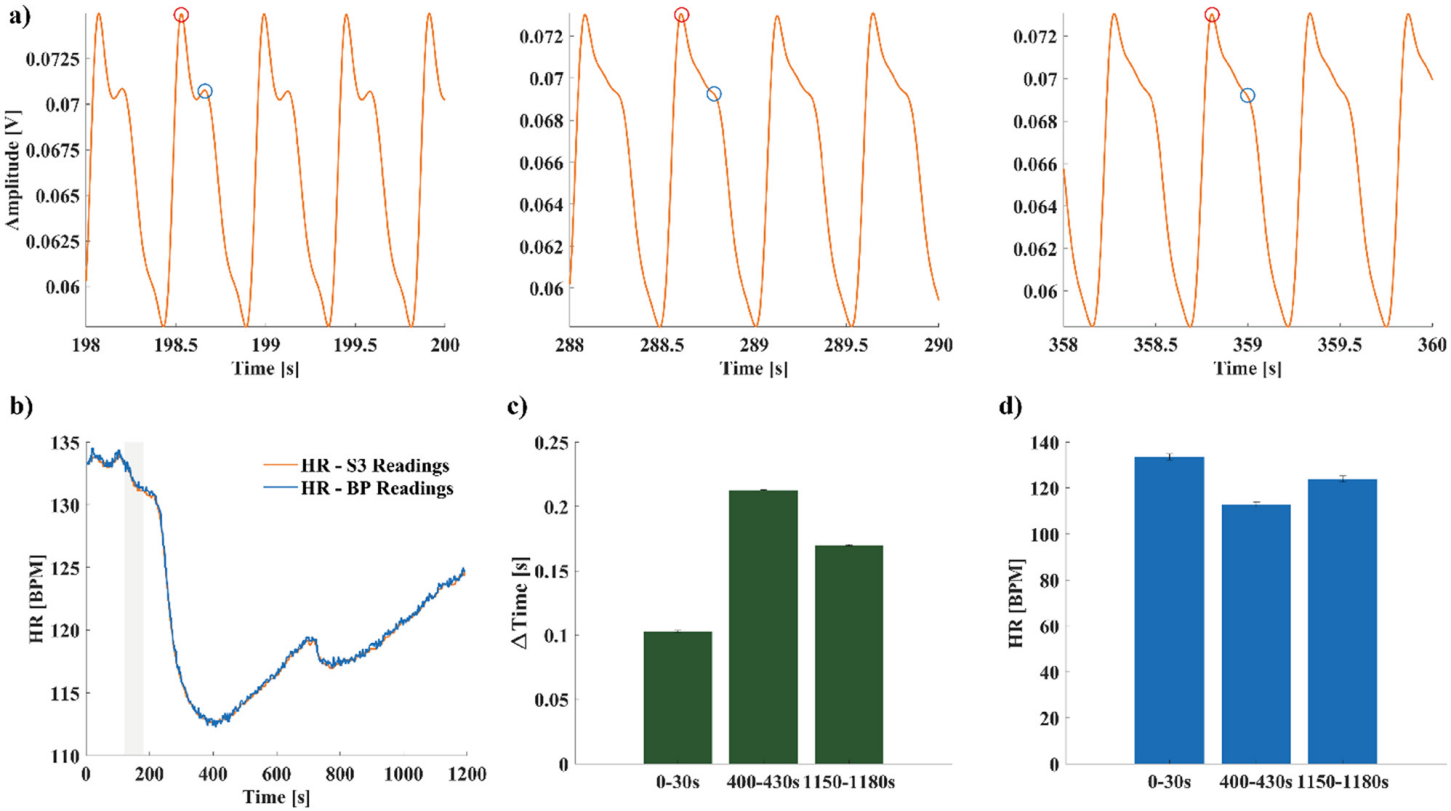
Esmolol pharmacological challenge. (a) S3 channel readings during different intervals of the pharmacological challenge. At baseline conditions, the waveform is bimodal, while when the HR decreases, the second peak (blue circle) becomes less prominent, reducing its amplitude. (b) HR computed using the S3 channel and the BP readings. The gray shaded area represents the duration of the esmolol injection. (c) ΔTime variations during 30 s windows in three different time intervals: 0–30 s (baseline), 400–430 s (HR minimum), and 1150–1180 s (return to baseline). (d) HR variations during 30 s windows in three different time intervals: 0–30 s (baseline), 400–430 s (HR minimum), and 1150–1180 s (return to baseline). We observed an inversely proportional relationship between ΔTime and HR.

As depicted in Fig. 2 and from Table II, the S3 channel produced the best results in terms of ABP similarity. As a result, we used S3 for the subsequent analysis. During AS1, we used two pharmacological stresses (esmolol injection, Fig. 3, and low-dose dobutamine injection, Fig. 4) to evaluate the sensor response to HR and ABP variations. As shown in Fig. 3(a), we analyzed the filtered S3 channel in different 2s windows to evaluate the modifications in MEMS sensor's signal shape caused by HR and ABP variations. During baseline conditions [before dobutamine injection, Fig. 3(b)], the shape is similar to what we observed in Fig. 2, with the presence of two peaks. When the HR and ABP increased, in turn, the second peak increased in amplitude [Fig. 3(a), right] from 70 to 79 mV, up to an increase to 91 mV, overcoming the first peak, while the distance between the two peaks decreased. We quantitatively evaluated this pattern by computing ΔTime during 30 s windows in three phases of the pharmacological challenge: baseline condition, plateau after drug injection, and return to baseline. We observed an inversely proportional relationship between ΔTime and HR [Figs. 3(c) and 3(d)], with ΔTime decreasing as HR increases. Starting from a ΔTime value 0.196 ± 8.500 × 10^−4^ s at baseline conditions (HR = 127.8 ± 1.4 BPM), it decreased by 50% of the original value (ΔTime = 0.102 ± 4.650 × 10^−4^ s) when the HR reached its maximum value (HR = 144.9 ± 1.5 BPM). During the return to baseline conditions, it increased again up to 0.178 ± 5.785 × 10^−4^ s coherently with HR decreasing (HR = 138.2 ± 0.9 BPM). We noticed a similar response during the esmolol injection challenge: ΔTime started from a value of 0.103 ± 8.835 × 10^−4^ during baseline conditions (HR = 133.5 ± 1.4 BPM), up to 0.212 ± 5.903 × 10^−4^ s when HR reached its minimum value (HR = 112.6 ± 1.3 BPM). Then, during the return to baseline, ΔTime decreased to 0.170 ± 5.205 × 10^−4^ s as HR increased (HR = 123.9 ± 1.4 BPM).

**FIG. 5.**
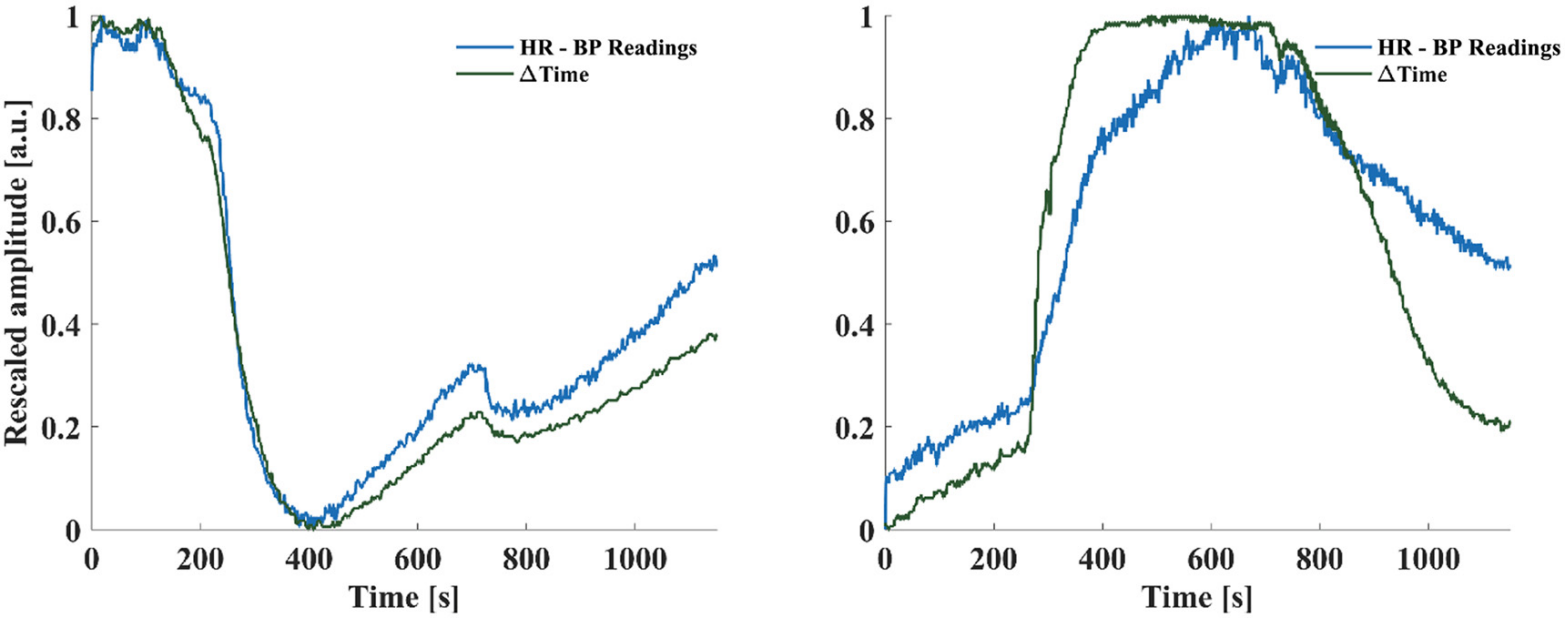
Comparison between ΔTime and HR (computed from BP readings) for correlation analysis. Both signals have been rescaled between 0 and 1 to analyze their behavior and compare their variations. From visual inspection and Pearson's correlation coefficient analysis, we found a strong linear correlation between the two signals (r = 0.9627 during esmolol challenge, r = 0.8805 during dobutamine challenge, and a.u.: arbitrary unit).

In addition to the qualitative evaluation of the relationship between ΔTime and HR in 30 s windows, we performed a correlation analysis between these two signals. We computed ΔTime for the overall challenge duration and then normalized both ΔTime and HR between 0 and 1 to compare them (Fig. 5). Then, we evaluated the linear correlation between the signals by computing the Pearson's correlation coefficient (r). By this analysis, we confirmed the high degree of correlation between these two signals for both challenges (r = 0.9627 for β-blockers challenge, and r = 0.8805 for dobutamine challenge).

### Results of chronic *in-vivo* experiments

**FIG. 6.**
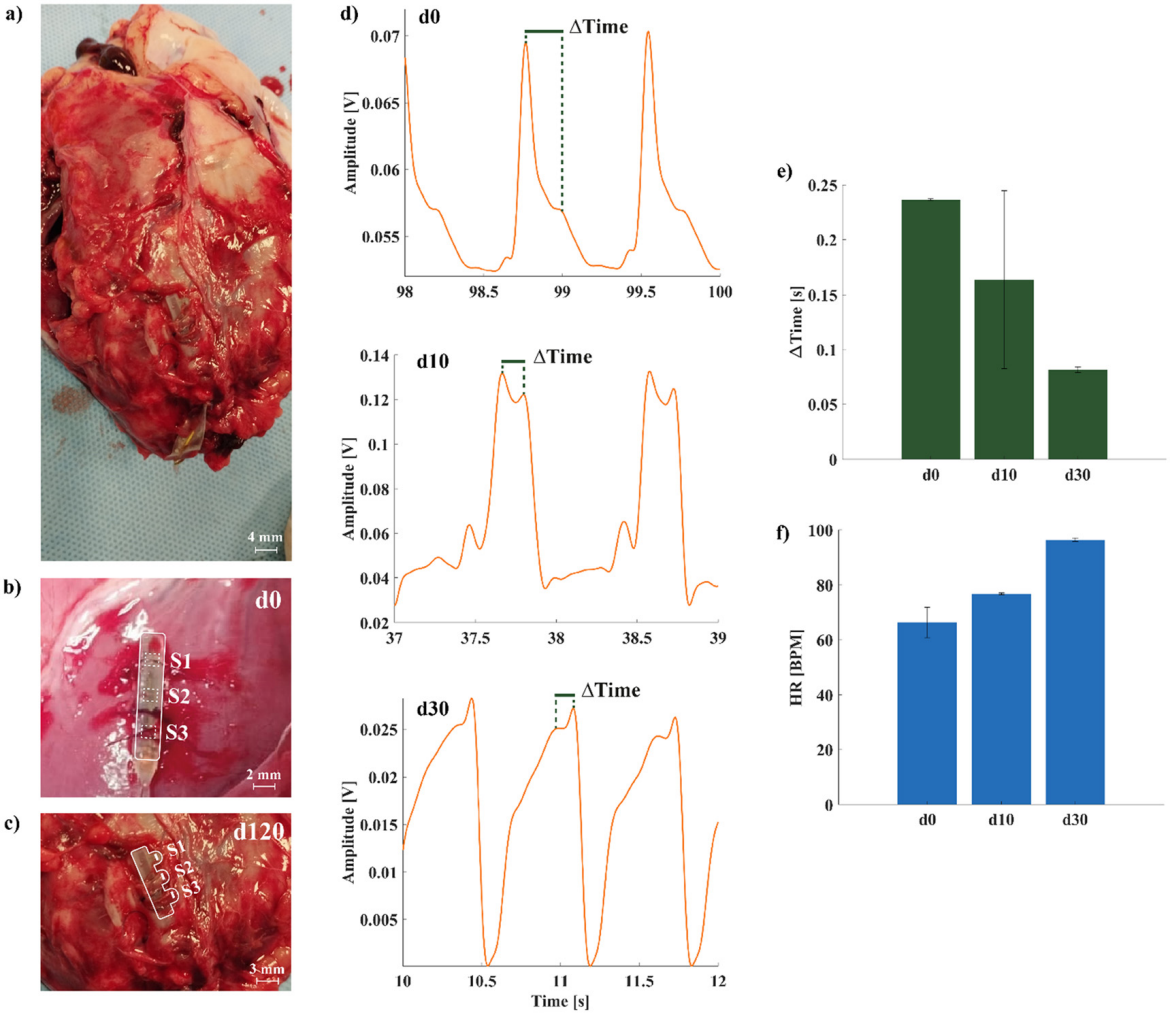
Chronic study results. (a) Heart explantation at d120. Near the heart apex, the broken cable residual can be seen in yellow. (b) Magnification of the MEMS sensor position at d0, with outline in white. The three sensors are implanted downward facing the heart muscle with mechanical stability guaranteed by the suture threads. (c) Magnification of the MEMS sensor position at d120, with outline in white. The MEMS sensor has rotated by 90°, with the three sensors establishing a looser contact with the heart surface. (d) S3 channel readings at d0, d10, and d30. The signal quality worsened during from d0 to d30, but it was always possible to retrieve HR information and ΔTime. The difference in amplitude between the signal at d10 and the signals at d0-d30 is due to an electrical failure of the external acquisition system. (e) ΔTime computed in 30 s windows at d0, d10, and d30. (f) HR computed in 30 s windows at d0, d10, and d30. As in acute experiments, we observed an inversely proportional relationship between ΔTime and HR. However, at d30, it was not possible to acquire the MEMS signal and the HR simultaneously due to a different experimental setup. The HR is recorded 2 min after the MEMS sensor acquisition.

Animal vital parameters are shown in Table III. We monitored the performances of the MEMS sensor during a 120-days chronic study by acquiring data in three different time points: d0, d10, and d30. We evaluated the status of the implanted sensor following the explant of the heart at d120 [Fig. 6(a)]. As shown in Figs. 6(b) and 6(c), the MEMS sensor orientation with respect to the heart muscle changed from d0 to d120; in particular, the three pillars rotated by 90°, establishing a looser contact with the heart surface. This rotation led to a worsening of the acquired signal quality, as it is possible to see in Figs. 6(d)–6(f). Degradation of the MEMS signal quality, however, did not affect the sensor capability to reliably compute the HR related to heart motion. We evaluated RMSE between the S3 channel of the MEMS sensor and the BP during 1-min acquisition at d10, obtaining RMSE = 1.8625. However, at d30, we were not able to acquire simultaneously the MEMS signals and the BP because of a different experimental setup. Thus, we compared the HR obtained from MEMS signals acquisition (HR = 94.1 BPM) with the HR obtained from the R-R interval from a successive ECG recording (HR = 94.6 BPM, 2-min distance between the recordings).

**TABLE III.** Vital parameters of chronic implanted animals (n = 2). Results are presented as mean ± std.

Parameter	d0	d10	d30	d120
HR (BPM)	65 ± 19	63 ± 21	79 ± 10	66 ± 5
SAP (mmHg)	125 ± 15	102 ± 17	101 ± 17	109 ± 28
DAP (mmHg)	69 ± 10	50 ± 27	50 ± 6	35 ± 6
MAP (mmHg)	87 ± 13	68 ± 11	67 ± 11	60 ± 13

Despite signal degradation, we were still able to compute ΔTime in 30 s windows, comparing it across the three different time points. We observed the same pattern found in acute tests with pharmacological challenges at d0, d10, and d30 [Figs. 6(e) and 6(f)] with ΔTime decreasing (d0 = 0.2364 ± 7.99 × 10^−4^ s, d10 = 0.1636 ± 0.08 s, and d30 = 0.0815 ± 0.03 s) as the HR increased (d0 = 66.4± 5.6 BPM, d10 = 76.8 ± 0.25 BPM, and d30 = 96.3 ± 0.6 BPM).

### Cardiac MRI

As shown in Table IV, the LVEF and the DT assessed by 1.5 T MRI in minipigs with implanted MEMS had comparable values to sham-operated minipigs. These data demonstrated that implantation of MEMS did not alter systolic and diastolic functions in the long-term. In fact, values remain within the range of normal function.

**TABLE IV.** Cardiac parameters by 1.5T cardiac magnetic resonance imaging (n = 2 animals without implanted MEMS, and n = 2 animals with implanted MEMS). HR, heart rate; SAP, systolic blood pressure; DAP, diastolic blood pressure; MAP, mean arterial pressure; LV EDV, left ventricle end-diastolic volume; LV ESV, left ventricle end-systolic volume; LV SV, left ventricle stroke volume; LV EF, left ventricular ejection fraction; DT, deceleration time; PFR-E, early peak filling rate; PFR-A, atrial peak filling rate; RV EDV, right ventricle end-diastolic volume; RV ESV, right ventricle end-systolic volume; RV SV, right ventricle stroke volume; and RV EF, right ventricle ejection fraction. The data are presented as mean ± standard error of the mean.

Cardiac MRI parameters	d30 without MEMS (n = 2)	d30 with MEMS (n = 2)
HR (BPM)	76 ± 18	63 ± 15
SAP (mmHg)	120.0 ± 4.5	102.0 ± 12.0
DAP (mmHg)	49.0 ± 16.5	50.5 ± 19.5
MAP (mmHg)	72.5 ± 12.5	68.0 ± 8.0
LV EDV (ml)	37.6 ± 2.9	57.2 ± 0.6
LV ESV (ml)	7.5 ± 2.6	15.3 ± 2.2
LV SV (ml)	30.0 ± 0.4	40.9 ± 37.7
LV EF (%)	80.0 ± 5.0	72.5 ± 4.6
DT (ms)	186 ± 60	153 ± 4
PFR-E (ml/s)	307.6 ± 33.6	304.85 ± 26.8
PFR-A (ml/s)	71.8 ± 10.1	51.8 ± 18.6
PFR-E/PFR-A ratio	4.4 ± 1.1	6.5 ± 1.8
RV EDV (ml)	55.1 ± 4.7	57.0 ± 4.0
RV ESV (ml)	23.3 ± 0.8	23.1 ± 0.3
RV SV (ml)	31.83 ± 3.87	33.95 ± 3.69
RV EF (%)	57.6 ± 2.1	59.4 ± 2.3

As shown in Table V, GLS values remained within the range of normal function and were similar in minipigs without and with MEMS at 30 days after surgery. Moreover, values of circumferential strain at implant site (mid anterior region) were similar to opposite site (mid inferior region) of the left ventricle in both experimental groups. In minipigs of both experimental groups, the analysis of delayed enhancement images did not reveal detectable extracellular collagen deposits, confirming the absence of possible ongoing remodeling.

**TABLE V.** Global and segmental left ventricular strain assessed by 1.5 T cardiac magnetic resonance imaging (n = 2 animals without implanted MEMS, and n = 2 animals with implanted MEMS). SAX, short-axis. The data are presented as mean ± standard error of the mean.

Left ventricular strain	d30 without MEMS (n = 2)	d30 with MEMS (n = 2)
Global longitudinal strain (LAX, %)	−23.05 ± 3.09	−23.265 ± 6.16
Basal anterior Circumferential strain (SAX, %)	−19.03 ± 10.17	−23.675 ± 2.03
Mid anterior Circumferential strain (SAX, %) (implant site)	−20 ± 3.45	−25.46 ± 2.36
Apical anterior Circumferential strain (SAX, %)	−24.34 ± 0.52	−27.335 ± 2.01
Basal inferior Circumferential strain (SAX, %)	−19.52 ± 5.2	−17.4 ± 6.78
Mid inferior Circumferential strain (SAX, %) (remote site)	−19.44 ± 3.30	−21.02 ± 3.91
Apical inferior Circumferential strain (SAX, %)	−26.27 ± 2.27	−25.05 ± 1.21

### Histological analyses

**FIG. 7.**
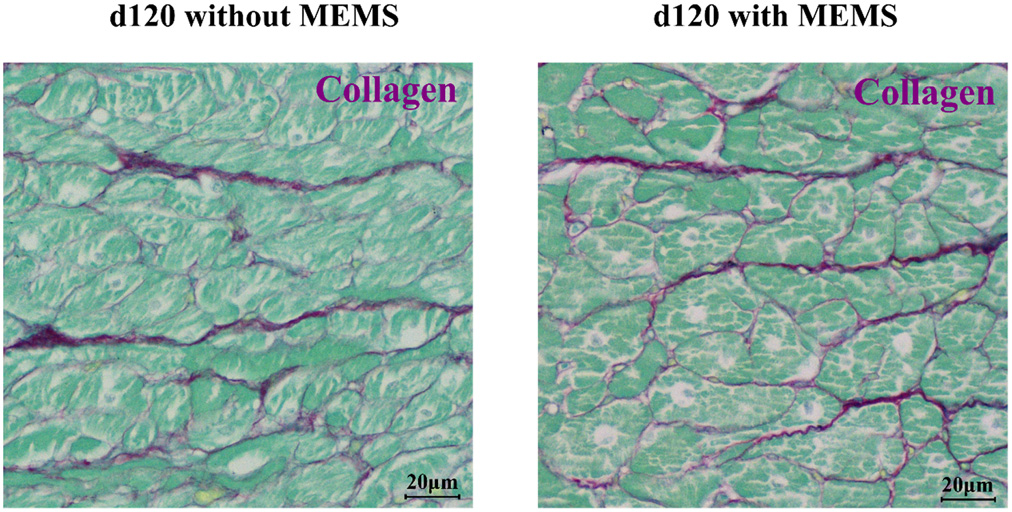
Histological analysis of the apical section (MEMS sensor implantation region) of the myocardial tissue post-sensor retrieval (at d120). The histology in the implanted (right) and control (left) animal shows no visible sign of tissue damage as it is possible to notice from a similar presence of collagen deposit (violet components) in the tissue.

As shown in Fig. 7, myocardial tissue of minipigs without and with implanted MEMS sensor in the apical region of implantation did not present any qualitative difference in collagen deposition. This histological analysis demonstrated that implantation of the MEMS sensor had no additional effect in terms of collagen deposition and tissue integrity.

## DISCUSSION

The findings of the present study demonstrate the capability of our piezoresistive cardiac MEMS sensor to consistently and accurately obtain heart rate (HR) readings. This was observed in benchtop tests as well as in acute and chronic scenarios involving healthy adult minipigs. These results indicate the potential suitability of the proposed sensor for closed-loop applications and provide a foundation for its future translation to human use for continuous postoperative and post-discharge monitoring of LV activity in patients undergoing cardiac surgery.

We compared the findings of our study with similar works from the state of the art implantable MEMS sensors for cardiac applications, highlighting the physical principle of sensing, the application, and the main findings of the studies. The results of this comparison are presented in Table VI.

**TABLE VI.** State of the art and commercial implantable MEMS cardiac sensors.

Work	Sensor type	Physical principle of sensing	Application	Main findings
Cong *et al.*^40^	Blood pressure monitoring	Capacitive	Acute studies on mouse	Resolution of 1 mmHg over 1 kHz bandwidth 3 dB noise degradation after implant
Nguyen *et al.*^39^	Cardiac motion monitoring	3-axis accelerometer	Acute studies on pigs	Device compliant with IEC 60601–1 Acceleration signals synchronized with ECG traces
Najafi *et al.*^36^	Blood pressure monitoring	Not available	Acute studies on dogs	High accuracy (<1 mmHg) compared to a Millar catheter sensor
Abraham *et al.*^42^	Pulmonary pressure monitoring	Capacitive	Human clinical trial	Reduction in the hospitalization rate (33%) and mortality (23%)
Steinhaus *et al.*^37^ Bourge *et al.*^38^	Intracardiac pressure monitoring	Capacitive	Human clinical trial	Lower rate of heart failure related events (21%), but results not significant
This work	Left ventricular activity monitoring	Piezoresistive	Chronic animal study	Sensor signal similar to intra-catheter ventricular signals High accuracy (RMSE < 2 BPM) compared to standard measures

The positioning and leverage of the poles were optimized using a test system, so that the readout of the sensor and the mechanical robustness were in accordance with the specifications. During the first surgical procedures, it was noted that the mechanical forces applied during implantation procedure were higher than initially anticipated. As a result, an additional mechanical support and improved encapsulation were developed to address the issue. We simulated the dynamics of the beating heart using a silicone phantom for appropriate benchtop tests. The waveform of the signal obtained from these tests is similar to what we observed during the *in vivo* sessions. We were able to reliably estimate the HR comparing it to the imposed reference signal, obtaining an RMSE of <1.5 BPM in eight over nine trials. During benchtop tests, the sensor was placed in a silicone hodge hooked to four points on the ventricle phantom to ensure a stable mechanical contact, mimicking the *in vivo* surgical procedure.

**FIG. 8.**
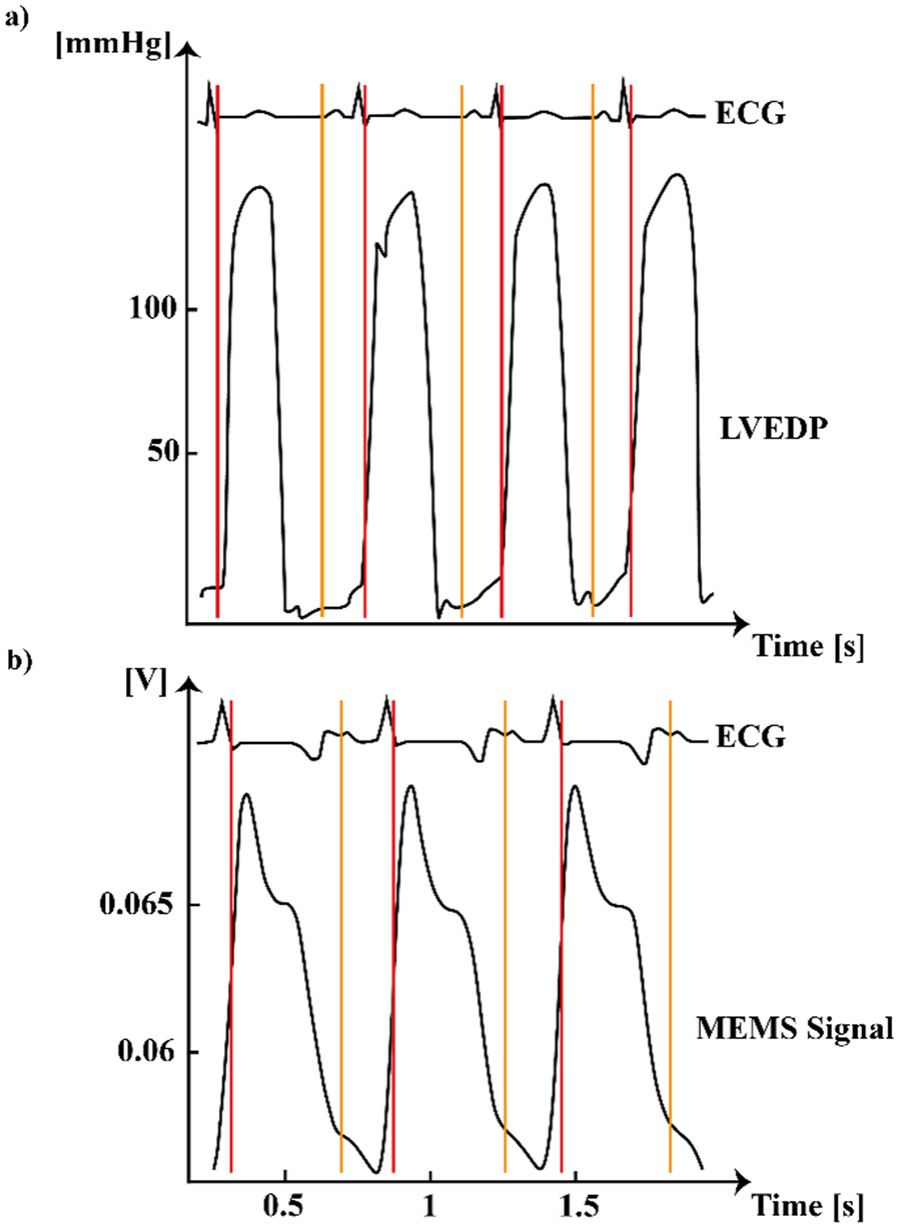
Comparison between the MEMS sensor signal and LVP from literature studies. (a) LVP (bottom signal) simultaneously synchronized with ECG (top signal), adapted from Ref. 49. (b) This study: MEMS sensor signal (bottom signal) simultaneously synchronized with ECG. The red lines indicate the beginning of the systolic phase, while the orange lines indicate the diastolic phase. In the two different studies, LVP and MEMS signal shapes are comparable and simultaneously synchronized with ECG variations, indicating the reliability of the MEMS signal to replicate invasive measurements of LVP. (a) Reproduced with permission from Dickinson *et al.*, Eur. J. Heart Failure **19**(11), 1483–1490. Copyright 2017 John Wiley and Sons.

During the *in vivo* sessions, we analyzed the waveform of the MEMS sensor from all channels comparing it to the ABP signal. We observed a clear similarity between the two signals in two out of three channels (S1 and S3), which coherently followed the different phases of the cardiac cycle. The difference between S1/S3 and S2 waveform could be due to the lower height of the S2 sensor. However, all the channels are able to detect the second positive peak of the signal, associated with the dicrotic notch (close of aortic and pulmonary valves^50,51^). The MEMS sensor's capabilities of reproducing typical features of the cardiac cycle empower its use for chronic cardiac monitoring, making it an alternative to sensors based on different physical principles, as acceleration or change in capacitance.^30,39^ Indeed, the waveform obtained from the epicardial MEMS sensor signal is directly related to the LVP signal.^8,48,52^ We compared what we observed in terms of the signal waveform from the MEMS sensor of the healthy heart with normal LVP pressure signals from previous studies,^49^ obtaining a qualitative match of the most important phases of the cardiac cycle (Fig. 8). For this reason, it may be used as a parameter for continuous monitoring of myocardial contraction stretch and stiffness during the cardiac cycle, typically acquired through a pressure catheter introduced into the left ventricle. The use of a ventricular pressure catheter is quite invasive and risky for the patient,^12^ and it is not possible to afford it in long-term studies due also to a higher risk of clots formation. We can assume that the use of our sensor implanted on the epicardial surface of LV is able to reproduce the waveform of an intraventricular pressure signal, allowing less invasive long-term monitoring. However, the comparison of synchronized signals shown in Fig. 8 is only qualitative, since we did not measure LVP using an intraventricular catheter. Further studies will be performed using quantitative metrics (i.e., correlation analyses) when the MEMS sensor and a left ventricular catheter will acquire data simultaneously. The possibility of having a quantitative match between LVP and our MEMS sensor signal could enhance the use of our sensor as a monitoring tool for LV wall stiffness and myocardial pump function for cardiac high-risk and/or pediatric patients. Further *in vivo* investigations are required to better support our claims. Future experiments will be aimed at LVP estimation over time at different loading conditions (preload and afterload) using our MEMS sensor, to evaluate its reliability.

The implantation procedure, inherently invasive, was designed to reduce impact with epicardial coronary arteries, to reduce the probability of sensor detachment from the LV wall and to limit fibrotic process entrapping and damaging the sensor. Indeed, the use of the sensor was devised to be implemented during cardiac surgery, for follow-up applications in high-risk patients. To reduce the risks associated with this kind of implantation procedure, the use of a pericardial patch graft as a cover for the MEMS sensor was considered. This approach was devised to keep the MEMS sensor in position and avoid possible artifacts due to micro-movements and fibrotic adhesions. Qualitative histological analyses (Sirius Red and Fast Green Staining) were performed to evaluate tissue integrity in the region of sensor implantation (close to the apex, Fig. 6). As shown in Fig. 7, the amount of collagen deposit between the implanted and control animal is similar, indicating the absence of fibrosis. To further confirm these findings, we analyzed GLS, which is more sensitive than LVEF in evaluating global contractile function of the left ventricle,^53^ and LV circumferential strain, a key function inherent in myocardial contractility of specific LV regions,^54^ and Gadolinium-delayed contrast-enhanced images, a standard procedure for assessing the presence of fibrosis in the myocardial wall.^55^ By comparing the results of the implanted group (n = 2) with the control group (n = 2), we observed no differences; thus, we were able to determine that there was no myocardial remodeling caused by the MEMS sensor implantation. However, the implantation procedure will be the subject of deeper analyses in future studies, since we were not able to retrieve high SNR signals from all the three channels of the MEMS sensor, probably due to an unstable mechanical contact.

Our approach enables the continuous extraction of signals that replicate the waveform of LVP, which is an invasive clinically relevant measurement. Indeed, LVEDP, a crucial diagnostic indicator of preload and ventricular stretch at the end of diastole, is directly measurable only through the invasive insertion of a micromanometer-tipped catheter into the left ventricle. Alternatively, it could be estimated through the measurement of the end-diastolic pulmonary artery wedge pressure through right heart catheterization. However, heart catheterization may expose to some patient-related and procedure-related complications and requires hospitalization.

We evaluated the time distance between the two peaks of the MEMS sensor's signal (ΔTime) and analyzed its variations during pharmacological stresses. We implemented two different pharmacological challenges: low-dose dobutamine injection for HR increase^56^ and esmolol injection for HR reduction.^57^ Our MEMS sensor proved reliable in computing the HR from all channels under both experimental conditions, despite differences in signal shape [Figs. 3(b) and 4(b), and Supplementary Material Figs. 2–5]. In all the three acute experimental studies (AS1, AS2, and AS3), we have obtained an RMSE < 2 BPM from all the sensor's channels. Based on the consistently low errors observed in both benchtop and *in vivo* experiments, we decided to utilize the signal from the S3 channels for further analyses. This is likely due to a more effective mechanical coupling between the S3 pillar and the beating surface (silicone phantom or heart muscle). It is possible that the S3 pillar remained securely fixed due to the weight of the electrical connection cable and the presence of suture threads, resulting in improved mechanical contact. Future iterations of the design will consider this behavior to fabricate a device able to consistently retrieve the same response from all channels. Overall, during the three acute experiments, we observed reliable MEMS sensor's readings immediately after positioning the sensor on the heart, indicating the sensor does not need a stabilization period to acquire signals.

In AS1, we analyzed the shape modification of the MEMS signal during the pharmacological challenges. During the low-dose dobutamine stress, we observed a huge increase in the second peak, overcoming the first peak, when the HR reached its maximum value [Fig. 3(a)]. Conversely, in short-term β-blockade induced by esmolol, we observed a decrease in the amplitude of the second peak, when the HR reached its minimum value, Fig. 4(a). Moreover, we evaluated HR and ΔTime in 30 s windows during three different phases of the pharmacological challenges: baseline conditions, HR increasing/decreasing, and return to baseline (washout). We observed an inverse relationship between HR and ΔTime variations: starting from the baseline conditions, when HR increased, ΔTime decreased (and vice versa). Additionally, we also observed a proportional relationship between these variables; ΔTime variations were, indeed, proportional to HR change [Figs. 3(c) and 3(d) and 4(c) and 4(d)], with greater ΔTime variations corresponding to higher HR increases or decreases during the challenges. Regarding the esmolol response, when HR was reduced by a ΔHR = 20.9 BPM (from baseline to minimum value), ΔTime was increased to 205% of its initial value, while when HR increased again up to ΔHR = 11.3 BPM [from minimum value to return to baseline), ΔTime was increased to 165% of its initial value [Fig. 4(c)]. We observed the same pattern and percentage variations during the low-dose dobutamine stress [Fig. 3(c)], indicating the invariance of the phenomenon to the direction and magnitude of HR variation.

Based on the data obtained through the ΔTime variations analysis, we conducted a quantitative correlation study between ΔTime variations and HR during all the experimental sessions with a pharmacological challenge. Both variables were normalized between 0 and 1 in order to ease their comparison. Figure 5 illustrates the similarity in variations between the two signals during the 20-min recording period for both challenges. Due to this high degree of similarity, we calculated the Pearson correlation coefficient between them, obtaining high values for both pharmacological challenges (r = 0.8805 for dobutamine challenge, and r = 0.9627 for the esmolol challenge). These findings suggest that the MEMS sensor is also able to provide information related to the heart cycle in an indirect way, paving the way for further investigation into its potential use in extracting heart-related information, such as mechanical stiffness and contractility. However, one of our current limitations is represented by not having explored multi-modal sensing. Indeed, in this first study, we only focused on the analysis of global LV activity parameters (HR, waveform of the signal). Future studies will focus on the simultaneous measurement of local and regional LV activity parameters, as regional contractility variations.

To assess the feasibility of utilizing the MEMS sensor for closed-loop applications, we conducted long-term testing by implanting the sensor in an adult minipig model for 4 months. The transcutaneous connector was placed under the skin of the animal, covered by medical tape for accessibility during the monitoring period for measuring purpose. We collected measurements at three different time points: d0, d10, and d30; however, we were not able to acquire useful signals at d120. Upon examining the explanted heart, we observed a 90° rotation of the MEMS sensor at d120 compared to its initial implantation position at d0. This rotation is illustrated in Fig. 6(c) (after heart explant) and Fig. 6(b) (implant location at d0). It is conceivable that fibrosis and subsequent dislocation of the sensor contributed to the deterioration of signal quality at d10 and d30, as depicted in Fig. 6(d). The weakened mechanical contact between the piezoresistive sensors and the heart surface may have resulted in an improper sensing configuration, with the heart muscle only contacting the side edges of the pillars. In future design iterations, modifications will be made to address the issue of rotation during long-term implants. One possible strategy to improve the stability of the MEMS sensor could involve suturing in different sections of the sensor to ensure a more secure mechanical contact to the beating heart. Additionally, the design can be modified to guarantee higher conformability to the heart surface while ensuring that its size remains compatible with the viability of epicardial branches of coronary arteries. We observed modification in the MEMS sensor's signal shape throughout the course of the experiment. At d10, the amplitude changed due to an electrical failure in the acquisition equipment, while at d30, the signal waveform was clearly altered. Nevertheless, we were still able to evaluate ΔTime at the three time points, computed in 30 s windows where the HR value remained stable. The inversely proportional relationship observed during acute experiments was maintained across d0, d10, and d30, highlighting the reliability of the measurements.

## CONCLUSION

In this work, we present a piezoresistive MEMS sensor design for cardiac surgery applications. We evaluated the sensor's performance in acute and chronic settings, through benchtop and *in vivo* testing in adult minipigs. The sensor consistently provided HR readings during acute sessions, despite a gradual decline in signal quality due to decreased mechanical contact with the epicardial surface. However, the signal waveform still represented the cardiac cycle features (systole and diastole peaks) necessary for HR computation. The results from the chronic study are particularly promising, as they indicate that the sensor can reliably compute a stable HR even after 1 month of implantation. The proposed design enables the potential acquisition of signals usually obtained from intraventricular devices, which are, nonetheless, more invasive and risky for the patients. By employing an innovative implantation method involving the use of a pericardial patch to cover the sensor, we could continuously monitor left ventricular activity for 30 days from a single sensor channel without altering the physiological cardiac cycle. In addition, post-sensor retrieval histological analyses highlighted no visible signs of damages in the implanted region (apical) of the myocardial tissue.

This finding suggests the potential for future investigations involving larger-scale animal testing, including comparison with ventricular pressure catheter. The ultimate goal would be to explore the feasibility of employing the MEMS sensor as a valuable tool for closed-loop applications, allowing for enabling long-term monitoring of cardiac activity in high-risk cardiac surgical patients. This study serves as a foundation for further research in this field, paving the way for advancements in sensor technology and potential improvements in high-risk patient care and monitoring.

## METHODS

### Sensor design and fabrication

**FIG. 9.**
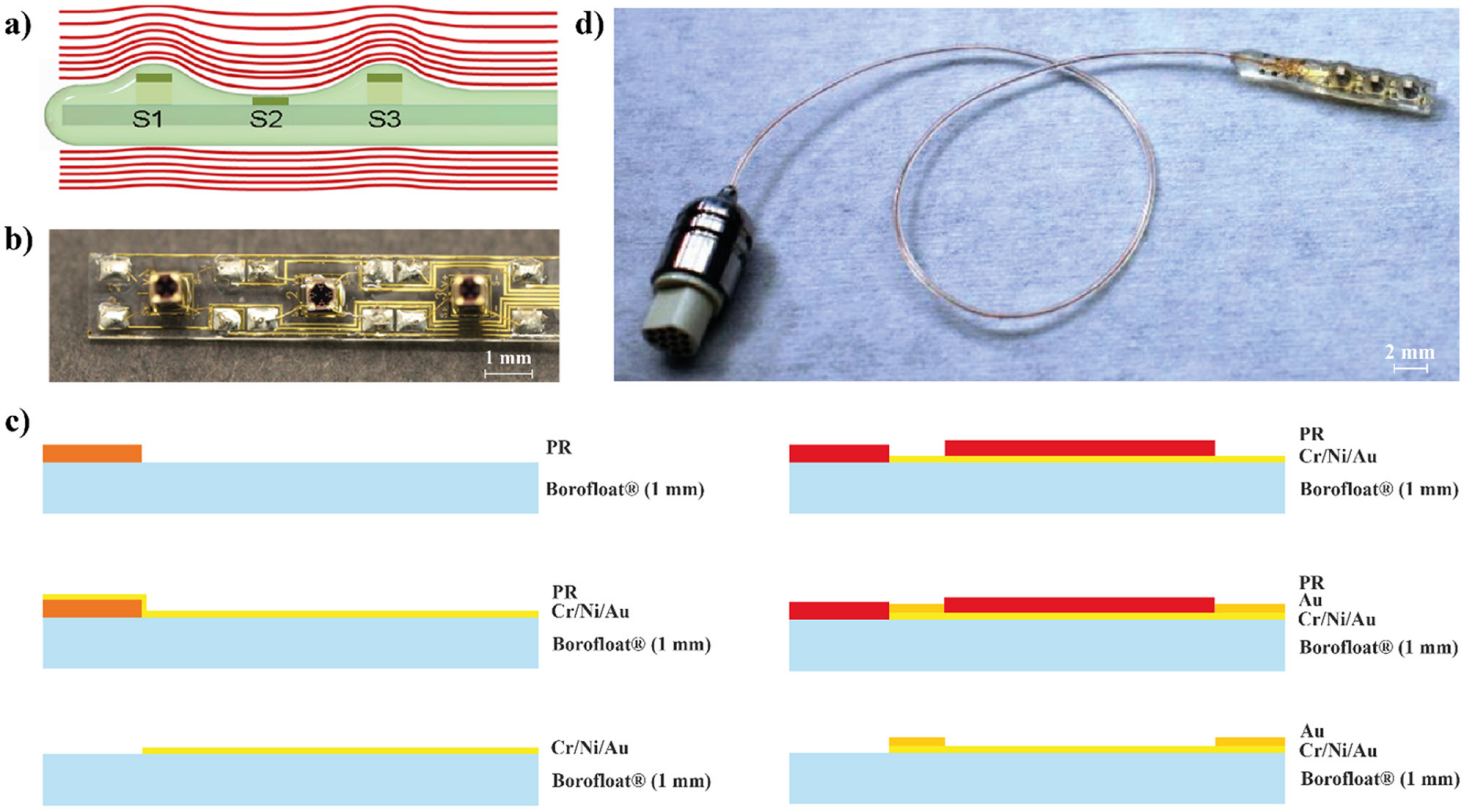
MEMS sensor design and fabrication. (a) Sensor concept: the three pillars (S1, S2, and S3) are designed to sense differences in pressure and retrieve cardiac cycle information. The red lines represent the pressure imposed by the beating heart. (b) Top view of an assembled prototype. Electrical contacting of the sensors was implemented by ultrasonic wire bonding. (c) Lithographic process for the fabrication of the MEMS sensor. (d) Completely assembled and encapsulated MEMS sensor.

The working principle of the MEMS sensor is depicted in Fig. 9(a). It is composed of three bare pressure sensor dies (Silicon Microstructure Inc., USA) equipped with four piezoresistive transducers placed at the edge of a pressure-sensitive membrane, and connected on-chip to a Wheatstone bridge. The platform where all the sensors are mounted has a footprint of 2 × 22.85 mm. The sensors are mounted on silicone pillars of different heights (2.625 mm lateral sensors S1/S3 and 2.425 mm middle sensor S2) able to detect forces imposed by the surrounding muscle tissue and discriminate different phases of the heart cycle. The pillars with the sensor dies on top were mounted upright on a 1 mm thick glass substrate and fixed with epoxy glue. The substrate was equipped with contact pads and conductive tracks realized by means of UV lithography, gold liftoff, and electroplating [Figs. 9(b) and 9(c)]. Electrical contacting of the sensor dies was done by ultrasonic wire bonding, and the opposite end of the bond wires was soldered to the contact pads on the carrier substrate. For power supply of the sensors and for connection with the readout electronics, thin enamel-insulated copper wires were soldered to the contact field at the rear end of the carrier substrate and then passed through a thin flexible tube made of medical grade PVC (Tygon Tubing, Saint-Gobain, France) to a miniature connector (Micro, Omnetics, USA). The solder joints on the carrier substrate, the wire ends, and the end of the PVC tubing were embedded with a UV-curable, acrylate-based resin (U305, Cyberbond, Germany) and thereby affixed on the substrate. Using a 3D-printed mold, the entire device was encapsulated in silicone (Dragonskin 10, Smooth-On, USA), covering the surface of the sensors with a layer about 200 *μ*m thickness. To prevent conceivable penetration of liquids into the Omnetics connector during the implantation, the connector was protected with a customized metal sheath and sealed with epoxy resin. A complete assembly of the sensor is shown in Fig. 9(d).

### Readout electronics

An analog-to-digital converter (ADC) board was realized through a commercial 16-channels (multiplexed), 24-bit ADC (ADS1258, Texas Instrument, USA). The detailed description of the circuit can be found in the Supplementary Material. The schematic of the circuit was implemented on a printed circuit board (PCB), and then a modular enclosure was developed and 3D-printed to contain the ADC board and to allow a straightforward connection through D-Sub connectors (SPI ports, sensor port, analog input ports). The ADC was connected to a Real-Time (RT) Embedded Target (myRIO 1900, National Instruments, USA) using 8 DIO lines implementing the SPI-compatible protocol with the ADC board.

Custom software was developed using the LabVIEW programming language to interface the ADC Board with the host computer used for data logging. This software is divided into two main parts: one running inside the RT Embedded Target enabling a remote interface to program and read the ADC and stream the collected data through a UDP stream, and another running on the host computer, listening for the data streamed from the RT Embedded Target.

### Sensor benchtop tests

To verify the correct functioning of the three force sensors, a small pressure chamber was fabricated, in which the sensor arrangement was exposed to variable pressures, to perform a calibration procedure. As a reference sensor, a pressure sensor (21S/80549.3, Omega Keller, USA) was used (supplementary material Fig. 1).

A pneumatic circuit with a silicone heart phantom (Dragonskin 10, Smooth-On, USA) was developed to test the behavior of the MEMS sensor in a simulated cardiac cycle [Supplementary Material, Fig. 2(a)]. The MEMS sensor was lodged in a polymer pocket hooked to the heart phantom [Fig. 1(a)]. A testing protocol with different pressures and the number of BPM application was established. For each experiment, a BPM sweep was performed (50–130 BPM) while keeping the imposed pressure fixed. Different values of the imposed pressure were tested (80, 100, and 120 mbar) to simulate different inflating conditions. The duty cycle of the driving signal was maintained at 30% to allow all the air inflated to escape during the deflation to avoid an excessive increase in the pressure inside the phantom heart. Data acquisition was performed in sessions lasting 60 s each, with 10 s of acquisition without air inflation, 40 s of air inflation, and again 10 s without air inflation. The sensor output, the number of beats per minute, duty cycle, pressure setpoint, and actual value were acquired and synchronized.

### Surgical preparation

**FIG. 10.**
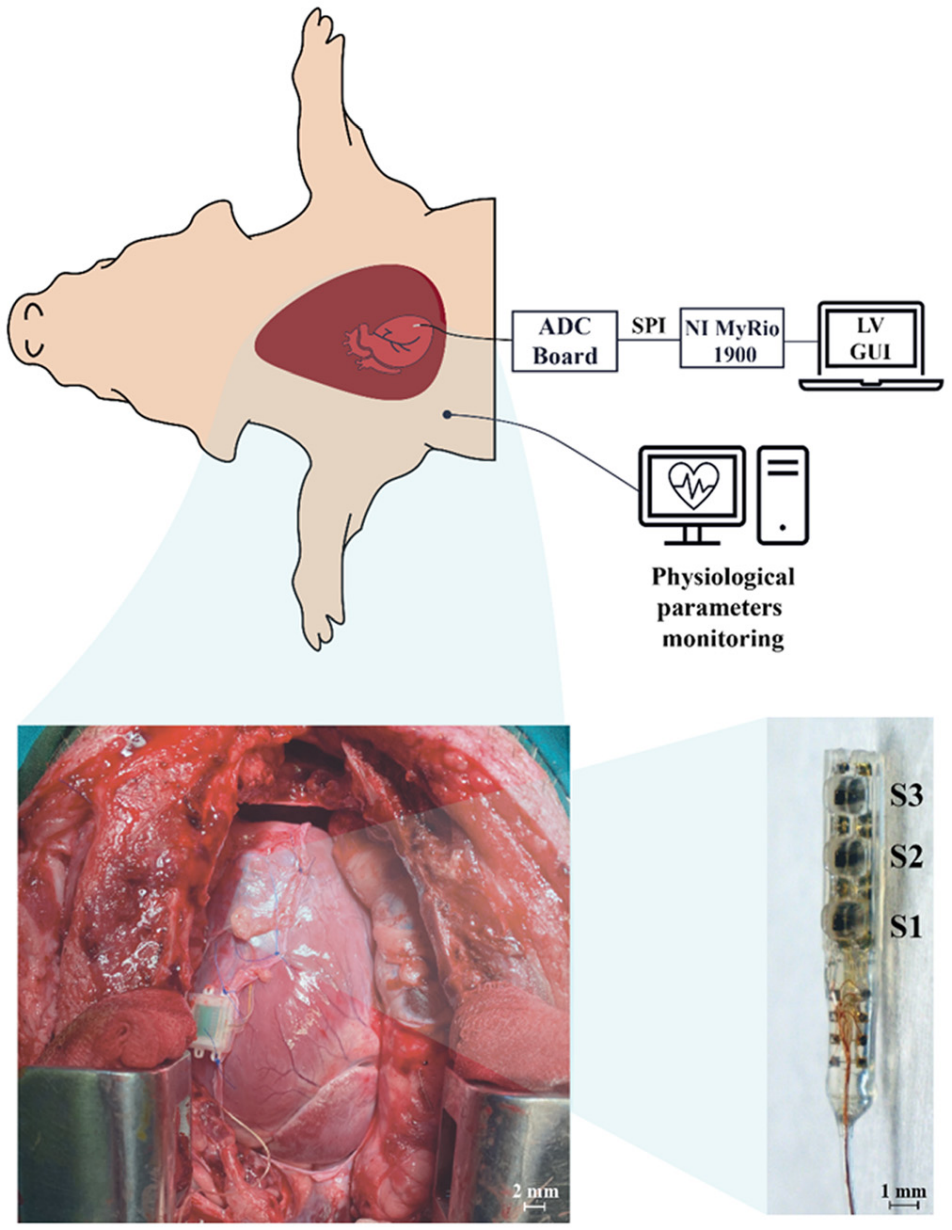
Experimental *in vivo* setup with heart and sensor magnifications. Once the heart is exposed from the thoracic cavity, the MEMS sensor is implanted facing S1–S3 downward, in close contact with the heart muscle, covered by a pericardial patch to guarantee better mechanical stability (bottom-left). Data acquisition is then performed using the same setup used for benchtop testing.

Four healthy adult male Göttingen minipigs (Ellegaard Göttingen Minipigs A/S, Dalmose, Denmark; avg body weight 35 kg) were enrolled in the present study. In order to define the feasibility, two acute and two chronic studies (1 month) were carried out. Animals were pre-medicated using Zoletil^®^ (10 mg/kg) and Stresnil (1 mg/kg). Each animal was anesthetized using Propofol (2 mg/kg intravenously) and maintained under 1%–2% sevoflurane in air enriched by 50% oxygen during mechanical ventilation.^11,25^ Throughout the experiments, the animal received an infusion of 500 ml NaCl (0.9%) solution to prevent dehydration. We performed a longitudinal incision followed by a sternotomy and pericardiotomy (Fig. 10). The two ends of the MEMS sensor were sutured to the anterior surface of the beating left ventricle with 5.0 nonabsorbable suture threads. An autologous pericardial patch graft was sutured to the healthy myocardium, covering the sensor to avoid its dislocation in the long-term due to chest wall and pleural adhesions. Once the sensor was safely implanted, it was connected to the readout electronics. The wire of the sensor was tunneled subcutaneously to the latero-cervical region, the chest was closed in layers, and the pneumothorax was reduced. Antibiotics were given after surgery, and the pigs were allowed to fully recover. The Omnetics connector was exposed only at the first experimental sessions to acquire data. To avoid fluid leakage inside the connector, a sterile plastic cap tightly adhered was used. Two age-matched healthy male Göttingen minipigs (avg body weight 35 kg) undergoing to sham surgery (sternotomy and pericardiotomy without MEMS sensor implantation) were used as control in the long-term feasibility study. Each animal was euthanized at d120 after surgery. The protocol for all animal studies (no. 76/2014 PR) was approved by the Italian Ministry of Health and was in accordance with the Italian law (D.lgs. 26/2014) and ARRIVE guidelines.

### Monitoring of cardiovascular parameters

Arterial blood pressure was measured via a fluid filled catheter inserted through the right femoral artery and attached to a P23ID strain-gauge transducer. The hemodynamic parameters were determined during one respiratory cycle and comprised the systolic (SAP) and diastolic (DAP) arterial pressure, the mean arterial pressure (MAP). Body temperature was maintained at 36.5–39 °C. We performed continuous electrocardiogram monitoring to evaluate cardiac rhythm. In clinical practice, parameters derived from femoral arterial pressure allow real-time assessment of LV contractility changes during different loading and inotropic conditions.^58^ In fact, LV end systolic pressure can be estimated from the femoral arterial pressure.^58^ Moreover, the invasive blood pressure signal is the most common signal measured on a beat-to-beat basis and may reflect heart rate measured by ECG.^59^ All data were recorded using the ADC board.

### Short-term feasibility study

We tested the sensitivity of the MEMS sensor to monitor cardiac activity at baseline and following administration of negative/positive inotropic stimuli during recording of ECG and ABP. After baseline recordings, we assessed the reliability of the signal measured by the MEMS sensor early after administration of low-dose dobutamine (10 *μ*g·kg^−1^·min^−1^ for 10 min), a beta-1 agonist used to quantify contractile reserve in minipigs.^55^ After a washout period of 10 min, we infused esmolol (bolus of 500 *μ*g and continuous infusion of 100 *μ*g·kg^−1^·min^−1^), a cardio selective beta1 receptor blocker with rapid onset.^11^

### Long-term feasibility study

The follow-up of the implantation was performed at three different time points, day 0 (d0), day 10 (d10), and day 30 (d30), during monitoring of ECG and ABP. On d10, the signals from the MEMS sensor were filtered using a 2 Hz cutoff frequency instead of 10 Hz, given the higher amount of noise compared to d0. At d30, ECG was used to monitor the animal status. For this reason, the performances of the MEMS sensor were compared to ECG changes. Animals' vital parameters were monitored throughout the 4-months (d120) period of the chronic study to evaluate their well-being.

### Cardiac magnetic resonance imagining (MRI)

1.5 T cardiac MRI was used to assess cardiac function in adult male minipigs at d30 after MEMS implantation (n = 2) and in adult male sham-operated minipigs at d30 after cardiac surgery (n = 2) in accordance with our previous studies.^55,60–62^ We measured the LV ejection fraction (LVEF, %), a noninvasive hallmark of systolic function, and the LV deceleration time (DT, ms), a hallmark of diastolic function resulting from the measurement of the time between the peak E velocity and the point, where the velocity returns to 0.^63^ The global longitudinal strain (GLS, %), an accurate and easily reproducible parameter obtained by 1.5 T cardiac MRI reflecting LV contractility,^64^ was calculated at basal, mid-cavity, and apical levels. It denotes the percentage of systolic myocardial deformation of the LV segments across the long axis. It was calculated by averaging the regional peak systolic values of all segments in all three planes. Short-axis cine images were used to measure the regional circumferential strain of the left ventricle. The epicardial and endocardial contours were traced manually, and papillary muscles were excluded. Technical imaging parameters were used as follows: repetition time (TR) = 4.1 ms, echo time (TE) = 1.8 ms, flip angle = 45°, measured voxel size = 1.9 × 1.9 × 8.0 mm^3^, and 30 cardiac phases. Regional LV circumferential strain (%), which is known to be altered within areas of myocardial scar,^65^ was measured from the base to the apex using the American Heart Association's (AHA) 17-segment model, a standardized segmental model that divides the left ventricle into specific regions^53^ in accordance with a previous study.^66^ In particular, the measurement of regional circumferential strain was also measured at the sensor implant site (mid anterior region) and opposite site (mid inferior region). To detect and quantify the myocardial fibrosis, late gadolinium delayed enhancement (LDE) was performed as previously described.^55^ Briefly, images were acquired in two-dimensional segmented inversion recovery-prepared gradient echo-sequence, 10 min after administration of contrast agent Gd-DTPA (0.2 mmol/kg iv), in short-axis views.

### Qualitative histological analysis of myocardial tissue

Following cardiac MRI, the animals were euthanized by injection of potassium chloride under general anesthesia, and the hearts were excised and cut into transverse (short-axis) thick slices from apex to base. Thus, we had three circular basal, mid-cavity, and apical short-axis slices of the left ventricle. The basal third was considered as the area that extends from the mitral annulus to the tips of the papillary muscles; the mid-cavity includes the entire length of the papillary muscles and the apical part considered as the part beyond the papillary muscles to the end of the cavity. Before specific staining, tissue samples underwent preparation through the following stages: fixation, processing, embedding, and sectioning. Tissue samples were fixed with 10% formalin. The tissue was cut in the microtome at thicknesses 5 *μ*m. From there, the tissue was mounted on a microscope slide for further steps of staining. Then, the paraffin slices have been deparaffinized in accordance with defined procedures. The deparaffinization was performed by xylene treatment (10 min, 2 times), followed by a series of washings with decreasing amounts of ethanol for tissue rehydration (100%, 90%, 75%, 50%, and 25%, 3 min twice for each ethanol). The slices were washed in tap water for 10 min. Sirius red and fast green staining were used to evaluate collagen deposition (9046, Chondrex, Inc.). The tissue samples underwent preparation through all stages according to the datasheet. Collagen fibers appeared magenta, while the non-collagen proteins were shown in green. Sections were acquired with a light microscope (DP20, Olympus).

### Data collection and analysis

Data from benchtop and *in vivo* tests were analyzed by comparing a reference signal to the MEMS sensor signal. All the analyses were performed using custom Matlab (Mathworks, Natick, MA, USA) software. The MEMS sensor's signals were acquired from the pneumatic circuit setup with a sampling frequency of 620 Hz from the ADC circuit in differential mode. The raw MEMS signals were digitally filtered with a fourth-order Butterworth low-pass filter with a 2 Hz cutoff frequency to obtain a single-mode waveform. Instantaneous heart beat frequency was computed by inverting the distance between successive peaks and then normalized (scaled by a factor of 60) to be expressed as BPM. The signals acquired from the three sensors on the MEMS sensor were compared to the BPM imposed by the pneumatic circuit by computing the root mean square error (RMSE) for each recording session, using the imposed BPM as a reference signal.

The data acquired from the MEMS sensor during the *in vivo* experiments were preprocessed by applying a fourth-order Butterworth low-pass filter, 10 Hz cutoff frequency. Arterial diastolic and systolic blood pressure (BP) were recorded from the ADC acquisition circuit for each heartbeat and then filtered with a fourth-order Butterworth low-pass filter with a 10 Hz cutoff frequency. ECG signal was processed using a notch filter to remove line interference and then smoothed using a moving average filter (n = 15 samples). Instantaneous HR was obtained from the blood pressure signal by computing the peak-to-peak interval under ECG monitoring,^67^ and it was used as a reference signal for the RMSE calculation. HR was also computed from the MEMS sensor's signals by inverting the distance between two successive diastolic peaks. Then, it was compared to the reference signal to evaluate the sensor's reliability in providing HR measurements. 1 min HR recordings were used to perform comparisons between the MEMS sensor's signals and the BP signal. Finally, before computing the RMSE, the two HR signals (obtained from the MEMS sensor and the BP signals) were median filtered (n = 10 samples) to remove outliers due to extrasystoles and ectopic beats. During both acute physiological challenges and chronic studies, the distance between the two peaks (ΔTime) of the MEMS signals was computed [Figs. 2(b) and 2(d)]. This parameter was analyzed to evaluate its relationship with HR variations.

## SUPPLEMENTARY MATERIAL

See the supplementary material for a detailed description of the readout electronics, together with a calibration procedure for the MEMS sensor. Furthermore, the setup design and the experimental protocol are indicated for all the benchtop tests performed with the pneumatic circuit. In addition, the Results section includes the measurements from the S1/S2 channels of the MEMS sensor during the pharmacological challenges, compared to physiological signals.

## Data Availability

The data that support the findings of this study are available from the corresponding author upon reasonable request.
